# Vacuum sealing drainage as an adjunct to surgical debridement in pediatric acute hematogenous osteomyelitis: a retrospective cohort study

**DOI:** 10.3389/fped.2026.1851350

**Published:** 2026-07-01

**Authors:** Zishuangyi Liu, Zhenjiang Liu

**Affiliations:** Department of Orthopaedics, Capital Center for Children’s Health, Capital Medical University, Beijing, China

**Keywords:** acute hematogenous osteomyelitis, negative pressure wound therapy, pediatric, surgical debridement, vacuum sealing drainage

## Abstract

**Background:**

Acute hematogenous osteomyelitis (AHO) in children can lead to chronic infection if not adequately drained. Vacuum sealing drainage (VSD) is widely used in adult osteomyelitis, but evidence in children remains limited.

**Methods:**

This retrospective cohort study included 55 consecutive children with AHO treated surgically between November 2019 and May 2025. Twenty-five patients underwent debridement plus VSD (VSD group), and 30 underwent debridement alone (control group). Baseline demographics, clinical characteristics, laboratory findings, and surgical details were collected. The primary outcome was length of hospital stay; secondary outcomes included number of surgical procedures, intravenous antibiotic duration, and infection recurrence within 3 months. Inverse probability of treatment weighting (IPTW) was applied to address confounding.

**Results:**

Before adjustment, VSD was associated with significantly longer hospital stay (33.2 ± 19.3 vs. 21.0 ± 9.5 days, *P* = 0.003) and a greater number of surgical procedures (3.6 ± 1.5 vs. 1.0 ± 0, *P* < 0.001) compared with controls. No significant differences were observed in intravenous antibiotic duration (*P* = 0.51), superinfection rate (*P* = 0.45), or 3-month recurrence (0% in both groups). After IPTW, all covariates were well balanced (SMD < 0.1), and the effect estimates remained essentially unchanged: VSD remained associated with longer hospitalization (mean difference: 10.1 days; *P* = 0.009) and more procedures (mean difference: 2.5; *P* < 0.001). All patients achieved infection control without progression to chronic osteomyelitis.

**Conclusion:**

In children with AHO, adjunctive VSD therapy was associated with significantly longer hospitalization and more frequent surgical interventions compared with conventional debridement alone, without measurable improvements in infection control or recurrence. These findings do not support the routine use of VSD in uncomplicated pediatric AHO and highlight the need for individualized patient selection.

## Introduction

Acute hematogenous osteomyelitis (AHO) is the most common invasive bacterial infection of bone in children, with an estimated annual incidence of 2–13 per 100,000 in developed countries and substantially higher rates in resource-limited settings (1, 2). Staphylococcus aureus, including methicillin-resistant strains (MRSA), accounts for 70%–90% of culture-positive cases (3, 4). If inadequately treated, AHO can progress to chronic osteomyelitis, leading to bone destruction, growth disturbance, pathological fractures, and long-term disability (5).

The cornerstone of successful management of pediatric AHO remains early diagnosis, prompt initiation of appropriate intravenous antibiotics, and timely surgical debridement when subperiosteal abscess or intramedullary pus is present (6, 7). Conventional surgical treatment consists of cortical window creation, curettage of necrotic tissue, and copious irrigation, followed by open packing or closed suction drainage (8). However, this approach has several drawbacks: it requires repeated painful dressing changes, prolongs hospital stay, increases the risk of nosocomial infection, and imposes significant psychological distress on both children and caregivers (9).

Vacuum sealing drainage (VSD), also known as negative pressure wound therapy, has emerged as a powerful adjunct in the management of complex acute and chronic wounds since its introduction by Fleischmann et al. in the 1990s (10). By applying controlled negative pressure to a sealed wound, VSD continuously removes exudate, reduces interstitial edema, promotes granulation tissue formation, and decreases bacterial colonization (11). Its efficacy has been well established in adult populations for conditions such as open fractures, diabetic foot ulcers, and postoperative wound infections (12–14).

Despite the theoretical advantages and widespread use of VSD in adults, its application in pediatric AHO remains limited and poorly characterized. To date, only a few small case series have reported favorable outcomes with VSD in children with AHO, with sample sizes ranging from 11 to 19 patients and lacking a control group (15, 16). Importantly, no internationally recognized clinical guidelines exist for the use of VSD in this population. The 2021 PIDS/IDSA clinical practice guideline for pediatric AHO does not provide recommendations regarding negative pressure wound therapy or VSD, reflecting the absence of high-level evidence. Consequently, decisions to apply VSD in pediatric AHO remain institution- and surgeon-dependent, with reported indications varying substantially across published case series. Thus, it remains unclear whether the addition of VSD to standard surgical debridement confers tangible benefits over conventional treatment alone in terms of infection control, hospital stay, number of surgical interventions, and complication rates. High-quality comparative evidence is urgently needed to guide clinical decision-making.

Therefore, we conducted a retrospective cohort study involving 55 consecutive pediatric patients with AHO treated at a single tertiary children's hospital. The aim of this study was to compare the clinical outcomes of surgical debridement combined with VSD vs. debridement alone, with particular focus on length of hospital stay, number of surgical procedures, intravenous antibiotic duration, and infection recurrence. We hypothesized that adjunctive VSD therapy would significantly shorten hospitalization and reduce the need for repeated debridement without increasing the risk of complications.

## Methods

### Study design and setting

This was a single-center, retrospective cohort study conducted at a tertiary children's hospital. The study was approved by the Institutional Review Board of CAPITAL CENTER FOR CHILDREN'S HEALTH, CAPITAL MEDICAL UNIVERSITY. For participants under the age of 16, informed consent was obtained from their parents or legal guardians. All procedures were performed in accordance with the ethical standards of the Declaration of Helsinki.

### Participants

We reviewed the medical records of all consecutive pediatric patients (aged ≤14 years) diagnosed with AHO and treated surgically at our institution between November 2019 and May 2025.

Inclusion criteria were: (1) age ≤14 years; (2) confirmed diagnosis of AHO based on compatible clinical presentation (fever, localized pain, swelling, warmth), elevated inflammatory markers [C-reactive protein [CRP] and/or erythrocyte sedimentation rate [ESR]], characteristic magnetic resonance imaging (MRI) findings (bone marrow edema, subperiosteal abscess, or intramedullary pus), and intraoperative findings of purulent material, with no sequestrum or necrotic bone, and with or without positive microbiological culture; (3) underwent surgical debridement with or without adjunctive vacuum sealing drainage (VSD); (4) complete medical records and at least 3 months of postoperative follow-up.

Exclusion criteria were: (1) chronic osteomyelitis defined as symptom duration >4 weeks, or presence of preoperative evidence (sequestrum, involucrum, or sinus tract) or intraoperative findings typical of chronic infection, including sequestrum, frankly necrotic bone separate from viable bone, or chronic fibrous granulation tissue. Superficial non-viable soft tissue or devitalized bone at the immediate margin of acute infection was not considered indicative of chronic osteomyelitis; (2) primary or secondary immunodeficiency; (3) prior surgical intervention at the same site; (4) concomitant bone tumor or metabolic bone disease; (5) incomplete data or loss to follow-up within 3 months.

### Treatment allocation and surgical procedures

Patients were allocated to either the VSD group or the control group based on the surgeon's preference and intraoperative assessment of infection severity. No formal randomization or allocation concealment was applied.

All patients underwent emergency surgical debridement upon admission. The procedure consisted of an incision over the area of maximal tenderness or fluctuance, blunt dissection through the muscle layers, adequate exposure of the affected metaphysis, and creation of a rectangular cortical window (approximately 0.5 cm in width, length tailored to the extent of purulent involvement). Pus and acutely inflamed soft tissue were evacuated, and meticulous curettage of purulent material, fibrinous exudate, and any loosely attached non-viable tissue at the inflammatory margin was performed. No sequestrum, necrotic bone, or chronic granulation tissue indicative of chronic osteomyelitis was observed in any patient. The surgical field was irrigated with 3% hydrogen peroxide solution (100 mL), 1:1,000,000 vancomycin-saline solution (500 mL), and copious normal saline (approximately 10,000 mL).

In the VSD group, after complete hemostasis, a vacuum sealing drainage system (InfoV.A.C.® Therapy Unit, KCI, San Antonio, TX, USA) was applied directly over the exposed bone surface and within the medullary cavity if necessary. The wound was sealed with an adhesive drape, and continuous negative pressure of −100 mmHg was maintained. The VSD dressing was changed every 3–7 days in the operating room under general anesthesia until the wound was clean, granulation tissue had formed, and there were no clinical signs of infection. Delayed primary closure or secondary closure was then performed.

In the control group, the wound was packed with iodoform gauze or left open with conventional non-adherent dressings after debridement and irrigation. Dressing changes were performed daily or every other day under aseptic conditions until the wound was deemed suitable for delayed primary closure or healing by secondary intention.

The decision to apply VSD was not randomized but was based on a combination of intraoperative findings and surgeon judgment. In our institution, during the study period, the following factors favored the use of adjunctive VSD: (1) extent of purulent involvement — diffuse intramedullary pus extending beyond a single metaphysis, or purulent material tracking into the adjacent soft tissue spaces; (2) soft-tissue condition—significant soft-tissue edema, impending or established soft-tissue compromise, or concern that repeated conventional dressing changes would be poorly tolerated or technically difficult; and (3) surgeon preference — individual surgeon's threshold for VSD use based on their training and prior experience. Conversely, VSD was generally not used when the infection was confined to a single metaphysis with a well-localized subperiosteal abscess, the overlying soft tissues were healthy, and a single debridement was expected to achieve adequate drainage. No formal scoring system or strict criteria were applied; the final decision was made intraoperatively by the attending surgeon. This description reflects real-world clinical practice and represents the source of potential confounding that we subsequently addressed using inverse probability of treatment weighting (IPTW).

### Antibiotic therapy

All patients received empirical intravenous vancomycin (15 mg/kg per dose every 8 h, adjusted according to renal function and therapeutic drug monitoring when available) immediately after diagnosis (i.e., before surgery in most cases). During the initial surgical debridement, intraoperative specimens (purulent material and/or bone tissue) were routinely obtained and sent for Gram staining, aerobic and anaerobic bacterial culture, and antimicrobial susceptibility testing. Deep tissue specimens were prioritized over swabs. Antibiotic therapy was subsequently de-escalated based on culture and susceptibility results, typically within 48–72 h after specimen collection. Intravenous antibiotics were continued until the patient was afebrile for at least 48 h and CRP levels had normalized or decreased by more than 50% from peak, a criterion supported by previous studies (17). Thereafter, oral antibiotics were administered to complete a total course of 3–4 weeks as recommended by the 2021 PIDS/IDSA guideline (18). The choice and duration of oral antibiotics were at the discretion of the treating physician.

### Outcome measures

The primary outcome was the total length of hospital stay (days), defined as the number of days from admission to discharge.

Secondary outcomes included: (1) number of surgical procedures (including the initial debridement and all subsequent VSD changes or re-debridement); (2) duration of intravenous antibiotic therapy (days); (3) incidence of superinfection (new pathogen isolated during treatment); (4) recurrence of infection within 3 months after discharge, defined as reappearance of local symptoms/signs, elevated inflammatory markers, and radiographic evidence requiring additional antibiotic or surgical intervention.

### Follow-up

All patients were scheduled for outpatient follow-up at 1 month and 3 months post-discharge. At each visit, clinical examination, serum CRP, and plain radiographs of the affected bone were obtained. Recurrence of infection, functional status, and any complications were recorded.

### Statistical analysis

Continuous variables were expressed as mean ± standard deviation (SD) or median with interquartile range (IQR) based on normality, and categorical variables as frequencies and percentages. Between-group comparisons in the original cohort were performed using Student's *t*-test or Mann–Whitney *U*-test for continuous variables, and chi-square test or Fisher's exact test for categorical variables, as appropriate.

To reduce confounding inherent to the non-randomized design, we conducted an inverse probability of treatment weighting (IPTW) analysis based on propensity scores. Propensity scores were estimated using a multivariable logistic regression model with treatment assignment (VSD vs. control) as the dependent variable. The model included the following prespecified covariates: age, sex, disease duration (days), affected bone (categorized as tibia, femur, fibula, calcaneus, and others), white blood cell count (×10^9^/L), C-reactive protein level (mg/L), and positive bacterial culture (yes/no).

Covariate balance before and after weighting was assessed using standardized mean differences (SMD); an absolute SMD < 0.1 was considered indicative of negligible imbalance. After IPTW, weighted means and percentages were calculated for baseline characteristics, and between-group comparisons were performed using weighted linear regression for continuous outcomes and weighted logistic regression for binary outcomes, both with robust sandwich variance estimators to account for weighting.

All statistical tests were two-sided, and a *P* value <0.05 was considered statistically significant. Analyses were performed using R version 4.2.0.

## Results

### Patient selection

**Figure 1:**
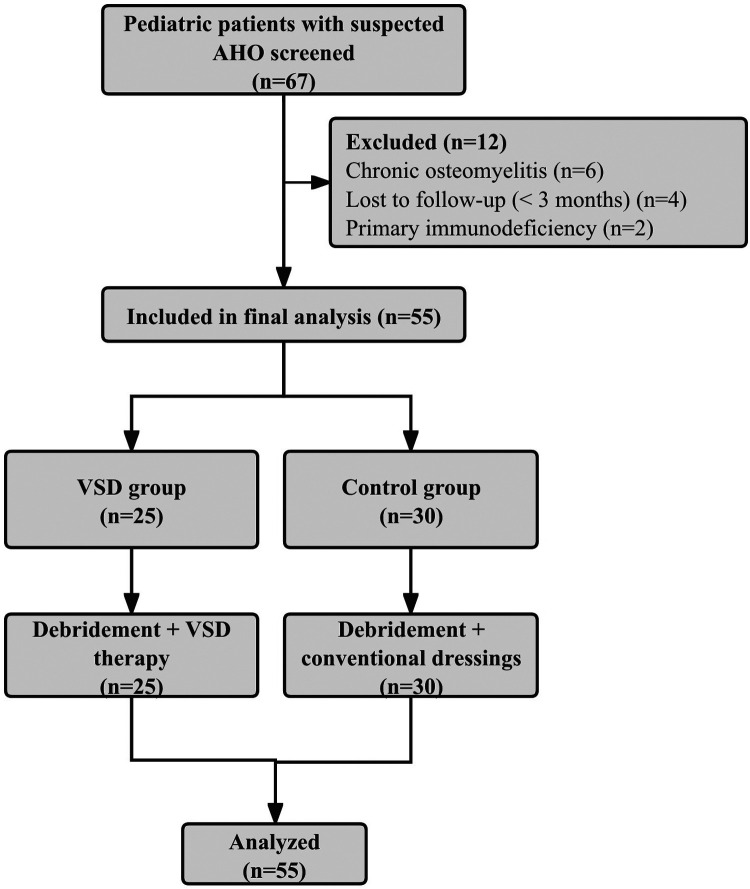
Flowchart of patient selection and group allocation.

A total of 67 consecutive pediatric patients with suspected AHO were screened between November 2019 and May 2025. After applying the exclusion criteria, 12 patients were excluded: 6 with chronic osteomyelitis, 4 lost to follow-up within 3 months, and 2 with primary immunodeficiency. Ultimately, 55 patients were included in the final analysis, of whom 25 underwent surgical debridement combined with vacuum sealing drainage (VSD group) and 30 received debridement alone with conventional dressing changes (control group). All 55 patients completed the 3-month follow-up assessment. The patient selection process is summarized in Figure 1.

### Baseline characteristics of the original cohort

Intraoperatively, no patient in either group exhibited features of chronic osteomyelitis, including sequestrum, necrotic bone, or chronic granulation tissue. The debrided material consisted primarily of pus, fibrin, and acutely inflamed soft tissue. The baseline demographic and clinical characteristics of the two groups are presented in Table 1. There were no statistically significant differences between the VSD group and the control group in terms of age (7.2 ± 3.1 vs. 6.5 ± 4.0 years, *P* = 0.48), sex (male: 56.0% vs. 66.7%, *P* = 0.42), disease duration (9.5 ± 4.7 vs. 8.2 ± 4.2 days, *P* = 0.29), white blood cell count (12.4 ± 5.6 vs. 12.3 ± 6.2 × 10^9^/L, *P* = 0.95), C-reactive protein level (54.3 ± 52.6 vs. 39.5 ± 34.6 mg/L, *P* = 0.22), or positive culture rate (56.0% vs. 36.7%, *P* = 0.15). Among the culture-positive cases, Staphylococcus aureus was identified in all 14 (100%) in the VSD group and in 9 of 11 (81.8%) in the control group (*P* = 0.22). Regarding antimicrobial susceptibility, methicillin-sensitive S. aureus (MSSA) accounted for 8/14 (57.1%) in the VSD group and 6/9 (66.7%) in the control group; methicillin-resistant S. aureus (MRSA) accounted for 6/14 (42.9%) and 3/9 (33.3%), respectively. All MRSA isolates were susceptible to vancomycin, linezolid, and trimethoprim-sulfamethoxazole. The remaining two culture-positive cases in the control group were Streptococcus pyogenes (*n* = 1) and Kingella kingae (*n* = 1). The detailed microbiological profile of intraoperative specimens, including culture positivity, pathogen distribution, and antimicrobial susceptibility, is summarized in Table 2.

**Table 1:** Baseline characteristics of pediatric patients with acute hematogenous osteomyelitis.

Variables	VSD group (*n* = 25)	Control group (*n* = 30)	*P* value
Age, years, mean ± SD	7.2 ± 3.1	6.5 ± 4.0	0.48^a^
Male, *n* (%)	14 (56.0)	20 (66.7)	0.42^b^
Disease duration, days, mean ± SD	9.5 ± 4.7	8.2 ± 4.2	0.29^a^
Affected bone, *n* (%)			
Tibia	9 (36.0)	8 (26.7)	0.46^b^
Femur	4 (16.0)	10 (33.3)	0.14^b^
Calcaneus	4 (16.0)	0 (0.0)	0.04^b^
Fibula	3 (12.0)	2 (6.7)	0.65^b^
Others	5 (20.0)	10 (33.3)	0.27^b^
WBC, ×10^9^/L, mean ± SD	12.4 ± 5.6	12.3 ± 6.2	0.95^a^
CRP, mg/L, mean ± SD	54.3 ± 52.6	39.5 ± 34.6	0.22^a^

Data are presented as mean ± SD or *n* (%).

a Student's *t*-test.

b Chi-square test or Fisher's exact test.

VSD, vacuum sealing drainage; WBC, white blood cell; CRP, C-reactive protein.

**Table 2:** Microbiological profile of intraoperative specimens.

Variable	VSD group (*n* = 25)	Control group (*n* = 30)	Total (*n* = 55)
Culture positivity, *n* (%)	14 (56.0)	11 (36.7)	25 (45.5)
Isolated pathogens (among culture-positive)			
Staphylococcus aureus, *n* (%)	14 (100)	9 (81.8)	23 (92.0)
MSSA, *n* (% of S. aureus)	8 (57.1)	6 (66.7)	14 (60.9)
MRSA, *n* (% of S. aureus)	6 (42.9)	3 (33.3)	9 (39.1)
Streptococcus pyogenes, *n*	0	1	1
Kingella kingae, *n*	0	1	1
Gram stain result (among culture-positive)			
Gram-positive cocci in clusters, *n* (%)	11/14 (78.6)	7/11 (63.6)	18/25 (72.0)
MRSA susceptibility (tested isolates)			
Susceptible to vancomycin, linezolid, TMPpathogens (among culture-SMX, *n*/*n* (%)	6/6 (100)	3/3 (100)	9/9 (100)

MSSA, methicillin-sensitive Staphylococcus aureus; MRSA, methicillin-resistant Staphylococcus aureus; TMP-SMX, trimethoprim-sulfamethoxazole.

Regarding the distribution of affected bones, the VSD group had a higher proportion of calcaneal involvement (16.0% vs. 0.0%, *P* = 0.04) and a lower proportion of femoral involvement (16.0% vs. 33.3%, *P* = 0.14), although the latter did not reach statistical significance. No significant differences were observed for tibia, fibula, or other sites (all *P* > 0.05). Overall, the two groups were generally comparable except for the imbalance in calcaneal osteomyelitis.

To further confirm the adequacy of infection control and to characterize the perioperative inflammatory response, we compared preoperative (admission) and postoperative (at the time of transition to oral antibiotics) laboratory and clinical parameters between the two groups. As shown in Table 3, both groups experienced significant reductions in CRP and WBC following surgical debridement and antibiotic therapy (all within-group *P* < 0.001). The mean time to becoming afebrile was 2.4 ± 1.1 days in the VSD group and 2.2 ± 0.9 days in the control group (*P* = 0.46). IV-to-oral switch, all patients in both groups were afebrile for at least 48 h, and CRP levels had decreased by more than 50% from peak or normalized. No significant differences were observed between groups in any of the measured parameters at either time point, consistent with the primary outcome analyses.

**Table 3:** Preoperative and postoperative changes in clinical and laboratory parameters.

Parameter	Time point	VSD group (*n* = 25)	Control group (*n* = 30)	*P* value (between groups at same time)
CRP (mg/L), mean ± SD	Admission (preop)	54.3 ± 52.6	39.5 ± 34.6	0.22
	At IV-to-oral switch	12.6 ± 15.3^a^	9.8 ± 12.1^a^	0.45
	Mean change (*Δ*)	−41.7 ± 48.2	−29.7 ± 31.5	0.28
WBC (×10⁹/L), mean ± SD	Admission (preop)	12.4 ± 5.6	12.3 ± 6.2	0.95
	At IV-to-oral switch	7.6 ± 2.8^a^	7.9 ± 3.1^a^	0.71
	Mean change (*Δ*)	−4.8 ± 5.2	−4.4 ± 5.6	0.79
Fever, *n* (%)	Admission	22 (88.0)	25 (83.3)	0.73
	Afebrile ≥48 h at switch	25 (100)^a^	30 (100)^a^	1.00
Time to afebrile (days), mean ± SD	–	2.4 ± 1.1	2.2 ± 0.9	0.46

CRP, C-reactive protein; WBC, white blood cell; preop, preoperative; IV, intravenous.

a *P* < 0.001 compared to admission value within the same group (paired *t*-test).

### Targeted antibiotic therapy and regimen duration

After culture and susceptibility results became available (typically within 48–72 h), antibiotic therapy was de-escalated to targeted regimens. For MSSA infections (14 patients), targeted therapy consisted of cefazolin (*n* = 12) or oxacillin (*n* = 2) intravenously. For MRSA infections (9 patients), vancomycin was continued as the targeted agent in all cases. No patient required combination therapy (e.g., vancomycin plus rifampin). The two non-S. aureus infections (Streptococcus pyogenes and Kingella kingae) were treated with intravenous penicillin G and ceftriaxone, respectively.

The total duration of intravenous antibiotic therapy was similar between the VSD group and the control group (13.5 ± 7.8 vs. 12.1 ± 8.9 days, *P* = 0.51). The total duration of the complete antibiotic course (intravenous plus oral) was also comparable (28.4 ± 6.5 vs. 27.8 ± 7.2 days, *P* = 0.74). The median time from surgery to transition to oral antibiotics was 11 days (IQR: 8–17) in the VSD group and 10 days (IQR: 7–15) in the control group (*P* = 0.38). Oral step-down therapy most commonly comprised cephalexin (for MSSA, 12/14 patients), clindamycin (for MRSA, 6/9 patients), or trimethoprim-sulfamethoxazole (for MRSA, 3/9 patients), with durations ranging from 14 to 21 days.

### Impact of VSD on antibiotic management

To determine whether the use of VSD independently influenced antibiotic therapy, we compared the above parameters between the two groups. No significant differences were observed in the timing of de-escalation, total intravenous antibiotic duration, total complete course duration, or the choice of targeted agents (all *P* > 0.05). These findings suggest that the addition of VSD did not alter antibiotic management decisions in this cohort.

### Treatment outcomes in the original cohort

Table 4 summarizes the treatment outcomes before adjustment. Patients in the VSD group had a significantly longer total hospital stay compared with the control group (33.2 ± 19.3 vs. 21.0 ± 9.5 days, *P* = 0.003). By study design, the VSD group underwent more surgical procedures (3.6 ± 1.5 vs. 1.0 ± 0, *P* < 0.001), as scheduled VSD changes were counted as separate operations. The duration of intravenous antibiotic therapy was similar between the two groups (13.5 ± 7.8 vs. 12.1 ± 8.9 days, *P* = 0.51). No patient in either group developed chronic osteomyelitis or experienced infection recurrence within 3 months post-discharge. One patient in the VSD group (4.0%) developed superinfection with Pseudomonas aeruginosa, which was successfully treated with targeted antibiotics; no superinfection occurred in the control group (*P* = 0.45).

**Table 4:** Treatment outcomes in the original cohort.

Outcome	VSD group (*n* = 25)	Control group (*n* = 30)	*P* value
Hospital stay, days, mean ± SD	33.2 ± 19.3	21.0 ± 9.5	0.003^a^
Total surgical procedures, mean ± SD	3.6 ± 1.5	1.0 ± 0	<0.001^a^
IV antibiotic duration, days, mean ± SD	13.5 ± 7.8	12.1 ± 8.9	0.51^a^
3-month recurrence, *n* (%)	0 (0)	0 (0)	–
Superinfection, *n* (%)	1 (4.0)	0 (0)	0.45^b^

a Student's *t*-test.

b Fisher's exact test.

IV, intravenous.

### Propensity score analysis and IPTW-adjusted outcomes

Propensity scores were successfully estimated for all 55 patients. After stabilization of IPTW weighting, the absolute SMD for all covariates were all less than 0.1, achieving an ideal balance. The baseline characteristics after weighting are shown in Table 5, and there were no significant differences between the groups (all SMD < 0.1). The weighted analysis showed that the length of hospital stay in the VSD group was still significantly longer than that in the control group (32.0 ± 19.3 vs. 21.9 ± 9.5 days, *P* = 0.009), and the number of surgeries was still higher (3.5 ± 1.5 vs. 1.0 ± 0 times, *P* < 0.001); there were still no statistically significant differences in the number of days of intravenous antibiotics (13.2 ± 7.8 vs. 12.3 ± 8.9 days, *P* = 0.61) and the rate of secondary infections (3.4% vs. 0.0%, *P* = 0.45). All children had no recurrence within 3 months. The weighted analysis results were consistent with the original analysis, suggesting that the results were robust.

**Table 5:** Baseline characteristics and outcomes after inverse probability of treatment weighting (IPTW).

Variables	VSD group (*n* = 25, weighted)	Control group (*n* = 30, weighted)	SMD	*P* value*
Age, years, mean ± SD	7.0 ± 3.1	6.8 ± 4.0	0.04	0.81
Male, % (weighted)	58.1	61.5	0.07	0.74
Disease duration, days, mean ± SD	9.2 ± 4.7	8.6 ± 4.2	0.08	0.68
Affected bone, % (weighted)				
Tibia	34.8	31.0	0.08	0.70
Femur	18.2	21.9	0.09	0.66
Calcaneus	8.0	5.2	0.09	0.63
Fibula	10.2	8.7	0.05	0.82
Others	28.8	33.2	0.09	0.64
WBC, ×10^9^/L, mean ± SD	12.4 ± 5.6	12.3 ± 6.2	0.01	0.94
CRP, mg/L, mean ± SD^a^	50.5 ± 52.6	44.8 ± 34.6	0.08	0.69
Positive culture, % (weighted)	52.6	41.0	0.09	0.65
S. aureus, % (weighted)^b^	52.6	33.8	0.08	0.70
Hospital stay, days, mean ± SD	32.0 ± 19.3	21.9 ± 9.5	–	0.009
Total surgical procedures, mean ± SD	3.5 ± 1.5	1.0 ± 0	–	<0.001
IV antibiotic duration, days, mean ± SD	13.2 ± 7.8	12.3 ± 8.9	–	0.61
3-month recurrence, % (weighted)	0	0	–	–
Superinfection, % (weighted)	3.4	0	–	0.45

VSD, vacuum sealing drainage; SMD, standardized mean difference; WBC, white blood cell; CRP, C-reactive protein; IV, intravenous.

Weighted means and percentages are presented. Standard deviations for continuous variables are the original SDs from the raw cohort. SMD < 0.1 indicates negligible imbalance after weighting.

*P* values derived from weighted linear regression (continuous variables) and weighted logistic regression (binary variables) using robust standard errors (sandwich estimator).

Stabilized IPTW weights were truncated at the 99th percentile to mitigate extreme values.

a CRP weighted means are IPTW-adjusted; SDs are the original raw SDs (VSD: 52.6, Control: 34.6).

b Weighted percentage of S. aureus corresponds to raw counts of 14/14 positive cultures in VSD and 9/11 in control group.

### Follow-up and complications

All 55 patients completed the scheduled 3-month follow-up. No patient developed chronic osteomyelitis, pathological fracture, or growth disturbance during the follow-period. Plain radiographs obtained at 1 and 3 months postoperatively showed progressive resolution of bone destruction and periosteal reaction, with no evidence of residual infection or sequestrum formation. No VSD-related adverse events (e.g., air leakage, dressing dislodgement, or vacuum failure) were recorded.

## Discussion

This retrospective cohort study evaluated the comparative effectiveness of adjunctive VSD vs. conventional debridement alone in children with AHO. After rigorous adjustment for confounding using IPTW, the VSD group demonstrated a significantly longer hospital stay and a greater number of surgical procedures compared with the control group. No significant differences were observed in intravenous antibiotic duration, superinfection rate, or 3-month recurrence. All patients achieved infection control without progression to chronic osteomyelitis. These findings suggest that the addition of VSD to standard surgical debridement in pediatric AHO does not confer superior infection-related outcomes but is associated with increased in-hospital resource utilization.

The longer hospitalization observed in the VSD group can be attributed primarily to the intrinsic characteristics of the VSD treatment protocol. Scheduled dressing changes were performed exclusively in the operating theater under general anesthesia at intervals of 3–7 days, and definitive wound closure was deferred until the wound bed was deemed adequately clean and granulated. This approach inherently prolongs the inpatient course. In contrast, most control patients underwent delayed primary closure within the first week or were discharged with open wounds managed by outpatient or home-based dressing changes, thereby truncating the index hospitalization.

Although IPTW achieved excellent balance across all measured covariates (all SMD < 0.1), residual confounding by unmeasured indicators of infection severity—such as the extent of medullary involvement, soft-tissue compromise, or virulence of the infecting organism cannot be entirely excluded. The higher baseline proportion of calcaneal osteomyelitis in the VSD group (16% vs. 0%), a condition associated with delayed healing and challenging soft-tissue coverage (19, 20), was successfully balanced after weighting (SMD = 0.09); nonetheless, confounding by indication may persist. Clinician bias toward more conservative discharge decisions in patients with an indwelling negative-pressure device may also have contributed to extended hospitalization.

The greater number of surgical interventions in the VSD group reflects the protocol-mandated serial debridement and VSD exchanges, each performed in the operating room. This finding does not represent treatment failure or complication; rather, it is an inherent trade-off the VSD strategy. In the control group, re-debridement was required only when clinical signs of inadequate drainage or persistent infection emerged. The absence of such events in the control group underscores the effectiveness of a single, well-performed debridement in most uncomplicated pediatric AHO cases.

Both groups received protocol-driven intravenous antibiotic therapy guided by predefined clinical and laboratory criteria (afebrile for ≥48 h and CRP normalization or ≥50% decline). This standardized approach likely explains the absence of between-group differences in intravenous antibiotic duration. The universal absence of recurrence at 3 months in both groups reflects the high baseline cure rate achievable with timely surgical drainage and appropriate antimicrobial therapy in contemporary pediatric AHO management (21, 22). Against this high benchmark, VSD offered no measurable advantage in preventing relapse or chronicity (16, 23).

Earlier case series without control groups reported favorable impressions of VSD in pediatric AHO, including shorter hospital stay and reduced need for repeated debridement (16, 24, 25). The discrepancy between those reports and the present study stems from several methodological differences. First, prior studies defined hospital stay as the interval from admission to VSD removal, whereas the present study measured total hospitalization until discharge—a more patient-centered and policy-relevant endpoint. Second, those series did not count VSD changes as surgical procedures, thereby underestimating the total operative burden. Third, the absence of a concurrent comparator precluded adjustment for secular trends or changes in adjunctive care. The present study, by including a contemporaneous control group and applying rigorous confounding control, provides more reliable estimates of VSD's incremental effects and challenges the assumption of its superiority in routine cases.

The findings do not support the routine use of VSD as an adjunct to surgical debridement in all children with AHO. In straightforward cases where infection is well-controlled after a single debridement, conventional treatment achieves excellent outcomes with shorter hospitalization and fewer operations. However, VSD may retain value in selected clinical scenarios: (1) extensive bone and soft-tissue destruction requiring staged reconstruction; (2) compromised soft-tissue envelopes where repeated conventional dressing changes would be technically difficult or intolerably painful; (3) failed primary debridement with persistent purulent drainage; and (4) resource-rich settings where the additional operative and hospital costs are acceptable to achieve a closed, granulating wound at discharge (10, 16, 26, 27). Shared decision-making with caregivers should explicitly address the anticipated trade-offs between inpatient burden and potential benefits.

From a health economics perspective, the longer initial hospitalization and greater operating room utilization associated with VSD carry substantial cost implications. Formal cost-effectiveness analyses in pediatric AHO are lacking; extrapolation from adult wound care suggests that VSD may be cost-neutral if it reduces readmissions, outpatient visits, and long-term complications (16, 28). In the present study, such offsetting benefits were not observed within the 3-month follow-up period. Therefore, the economic case for routine VSD in this population remains unproven.

In this study, open wound management with packing or conventional dressings was used as the control condition. This primarily reflected the established standard practice at our institution for pediatric acute hematogenous osteomyelitis patients who did not receive vacuum sealing drainage during the study period (November 2019—May 2025). Primary closure with a conventional closed-suction drain (e.g., Penrose or Jackson-Pratt) was not routinely performed at our center for this condition, and the number of patients managed with that approach was too small to constitute a separate comparison group. Open packing after debridement is a well-recognized technique in orthopedic infection surgery, allowing passive drainage and dead space obliteration without indwelling foreign material, and to the best of our knowledge, no randomized controlled trial has demonstrated superiority of primary closure with a drain over open management in this setting. After infection control is achieved (afebrile for ≥48 h, declining C-reactive protein, and a clean wound base), caregivers can be trained to perform home dressing changes—a feasible strategy under appropriate conditions. Nevertheless, open wound management has inherent disadvantages, including pain during dressing changes, risk of secondary contamination, and increased caregiver burden. Consequently, our findings do not directly inform the comparison between vacuum sealing drainage and primary closure with a drain; future prospective studies, particularly randomized controlled trials, should consider including a closed-suction drainage arm as a clinically relevant comparator to both vacuum sealing drainage and open wound management.

An important secondary question is whether adjunctive VSD influenced antibiotic management, such as the timing of de-escalation, total duration of intravenous therapy, or choice of targeted agents. In this cohort, we found no significant differences between the VSD and control groups in any of these parameters. The time from surgery to transition to oral antibiotics was comparable (median 11 vs. 10 days, *P* = 0.38), as were total intravenous and complete course durations. This suggests that, in the context of a protocol-driven antibiotic strategy (afebrile ≥48 h plus ≥50% CRP decline from peak), the addition of VSD did not alter the pace of infection control or the clinician's decision to step down therapy. These findings reinforce the conclusion that VSD does not confer an added advantage in achieving infection resolution, at least as reflected by antibiotic de-escalation criteria.

An important consideration in interpreting our findings is the absence of established clinical guidelines for VSD application in pediatric AHO. The 2021 PIDS/IDSA guideline for pediatric AHO makes no recommendation regarding negative pressure wound therapy, and most published studies are case series without control groups. Consequently, the decision to use VSD remains surgeon-dependent, leading to potential selection bias and confounding by indication—a limitation inherent to retrospective comparisons of this kind. In our cohort, VSD was applied selectively based on intraoperative assessment of infection severity (e.g., extent of purulent involvement, soft-tissue condition) rather than a standardized protocol. While we employed IPTW to adjust for measured confounders, unmeasured factors such as the precise extent of bone destruction, surgeon's threshold for VSD use, and soft-tissue compromise may still have influenced treatment allocation. Our findings should therefore be interpreted as reflecting real-world practice at a single institution rather than a definitive comparison under strictly controlled conditions. Future prospective studies and randomized trials are needed to establish standardized indications for VSD in pediatric AHO.

Several limitations must be acknowledged. First, the retrospective, non-randomized design is susceptible to selection bias and residual confounding despite advanced statistical adjustment. Unmeasured confounders, such as the precise extent of bone destruction, nutritional status, pathogen virulence, and surgeon's threshold for VSD use may have influenced both treatment allocation and outcomes. Second, this is a single-center study, which may limit generalizability; institutional practices regarding VSD indications, perioperative care, and discharge criteria may differ elsewhere. Third, the modest sample size, although larger than any previous series, provided limited statistical power to detect small differences in rare events (e.g., recurrence, serious adverse events). Fourth, the 3-month follow--up is insufficient to assess late complications such as growth disturbance or chronic osteomyelitis; longer surveillance is warranted. Fifth, patient-reported outcomes (pain, quality of life, caregiver burden) and functional outcomes (range of motion, return to normal activities) were not measured, yet these are arguably more relevant to patients and families than process metrics like hospital stay. Sixth, the control group was limited to open wound management; primary closure with a conventional drain was not evaluated. Whether vacuum sealing drainage offers any advantage over closed-suction drainage in pediatric acute hematogenous osteomyelitis remains unknown. Additionally, open wound management itself has inherent limitations, including pain during dressing changes, risk of secondary infection, and increased caregiver burden, which were not directly measured in this retrospective study. Finally, the absence of a formal cost-effectiveness analysis limits the ability to inform resource allocation decisions.

A multicenter randomized controlled trial is needed to definitively establish the role of VSD in pediatric AHO. Such a trial should stratify by disease severity, standardize VSD and antibiotic protocols, and incorporate a comprehensive set of outcomes including clinical efficacy, patient-reported experience, functional recovery, and health economic evaluation. In the absence of trial data, well-designed prospective cohort studies using target trial emulation frameworks could provide valuable complementary evidence. Additionally, research should focus on optimizing VSD parameters—negative pressure level, cycling mode, change frequency—and identifying patient subgroups most likely to derive benefit.

## Conclusion

In this retrospective cohort of children with acute hematogenous osteomyelitis, adjunctive vacuum sealing drainage was associated with significantly longer hospitalization and more surgical procedures compared with conventional debridement alone, without measurable improvements in infection control, antibiotic duration, or recurrence. These findings indicate that VSD is not inherently superior to traditional management in routine pediatric AHO and should be reserved for selected complex cases where its potential benefits outweigh the increased inpatient burden. Pending higher-quality evidence, clinical decision-making should be individualized and grounded in transparent discussion of the expected trade-offs. Our findings apply specifically to comparisons between vacuum sealing drainage and open wound management; direct comparisons with primary closure using conventional drains are needed in future studies. In addition, future research should focus on establishing standardized clinical indications for VSD in pediatric AHO to guide patient selection and improve the interpretability of comparative studies.

## Data Availability

The original contributions presented in the study are included in the article/Supplementary Material, further inquiries can be directed to the corresponding author.
